# Flexural Strength and Morphological Study of Different Multilayer Zirconia Dental Materials

**DOI:** 10.3390/ma17051143

**Published:** 2024-03-01

**Authors:** Andrea Labetić, Teodoro Klaser, Željko Skoko, Marko Jakovac, Mark Žic

**Affiliations:** 1 University of Zagreb School of Dental Medicine, Gunduliceva 5, 10000 Zagreb, Croatia; andrea.labetic@gmail.com; 2 Ruder Boskovic Institute, P.O. Box 180, 10000 Zagreb, Croatia; klaser@irb.hr; 3 Department of Physics, Faculty of Science, University of Zagreb, Bijenicka, c. 32, 10000 Zagreb, Croatia; zskoko@phy.hr

**Keywords:** multilayer zirconia, flexural strength, ZirCad Prime, Cercon ht ML, Katana ZIRCONIA YML

## Abstract

Nowadays, yttria (Y^3+^)-stabilized ZrO_2_ (Y-TZP) is the most commonly used material in dental prosthetics. Y-TZP dental ceramics are mainly stabilized via the addition of 3 mol% yttrium oxide (Y_2_O_3_). These ceramics exhibit excellent mechanical properties, including high flexural strength, fracture toughness, elastic modulus, etc. Some manufacturers have recently introduced a new class of dental materials with multilayer composition with the aim of combining the advantages of adding more or less Y_2_O_3_ to the ceramic composition in one Y-TZP material. The flexural strength values of multilayer Y-TZP may vary depending on the dimensions of the specimen, layer distributions, and especially the layer exposed on the maximum tension side, i.e., loading configuration. Although previous studies have examined the flexural strength of separate Y-TZP layers, capturing the flexural strength of multilayer Y-TZP is still challenging. However, one should keep in mind that multilayer flexural strength is important for clinical indications. The objective of this study is to compare the flexural strength of three distinct multilayer translucent Y-TZP materials made up of layers with different Y^3+^ contents. Rectangular samples (2 mm × 2 mm × 16 mm) were prepared from CAD/CAM discs using the milling machine Programill PM7 (Ivoclar Vivadent AG). Milled bars were tested for flexural strength in a three-point bending test (ISO 6872:2015) using a universal testing machine (Inspekt Duo 5kN; Hegewald & Peschke, Nossen, Germany) at a crosshead speed of 0.5 mm/min. Representative samples of each type of material were selected for quantitative and qualitative analysis of the microstructure. Representative samples of each type of material were selected for structural, mechanical, and microstructural analyses.

## 1. Introduction

Zirconia (ZrO_2_) is a versatile and durable ceramic material that has gained widespread acceptance in dental applications [1,2,3,4] due to its exceptional properties, including high flexural strength, fracture toughness, and dimensional stability [5]. These properties are of utmost importance for the application of ZrO_2_-based materials in dentistry. ZrO_2_ exists in three different phases: monoclinic (m), tetragonal (t), and cubic (c). At room temperature, pure zirconia is in the monoclinic phase. However, when heated, it transforms into the tetragonal phase. 

The tetragonal zirconia phase (TZP) can be stabilized at room temperature by incorporating yttria (Y^3+^) as a stabilizer [5,6]. The presence of the t-ZrO_2_ phase yields improved mechanical properties that are vital for dental applications. This type of ZrO_2_-based material is known as yttria-stabilized tetragonal zirconia polycrystals (Y-TZP), and it is commonly used as a structural ceramic [1,2,7]. The same stabilizing effect can also be achieved by adding, e.g., Mg^2+^, but the resulting Mg-TZP ceramics exhibit inferior strength and fracture toughness compared to Y-TZP [8].

Y-TZP is especially well-suited for restorative dentistry due to its chemical and mechanical stability, coupled with high mechanical strength [1,9]. Moreover, the latest generation of ZrO_2_-based materials, which are composed of both 3Y-TZP (i.e., the addition of 3 mol% Y^3+^) and 5Y-TZP (i.e., the addition of 5 mol% Y^3+^) in a single disc, are now being called universal materials for all clinical indications. This includes applications ranging from anterior single crowns to full-arch restorations [10]. 

Recently, the new generations of monolithic zirconia ceramic systems have also increased the Y^3+^ content to approximately 4 and 5 mol%. These ceramics systems contain a higher portion of c-ZrO_2_ polycrystals, which contribute to enhanced optical properties [11]. A type of this ceramics system is called “multilayer” as it has layers of polychromatic and translucent zirconia that produce a gradient effect, ranging from enamel to dentin shades in the single disc before milling. Please note that these multilayered zirconia materials can be used for both posterior and anterior restorations [12].

Since dental ceramics are brittle materials, their mechanical properties play a crucial role in preventing crack propagation and ensuring the long-term durability of restorations. These ceramics are more prone to cracking under tensile stress [13]. The mechanical properties that should characterize ceramics in the long term are flexural strength and fracture toughness. Furthermore, dental materials, including crystallized silica-based glass-ceramics [14] and polymer-infiltrated ceramic network (PICN) composites [15], undergo routine flexural strength testing in both research and clinical settings. One may observe that flexural strength can be determined with a three-point or four-point bend test or a biaxial flexural test [13].

Another advantage of Y-TZP as the material for dental applications is compatibility with computer-aided design (CAD) and computer-aided manufacturing (CAM) technology [1,2,9,16,17,18,19,20,21]. With the advent of CAD/CAM technology, Y-TZP materials have become the material of choice for the fabrication of high-quality and all-ceramic dental restorations [4,22,23]. In addition, the application of CAD/CAM technology, which facilitates the shaping of this material in numerous ways, promotes the development and investigation of diverse Y-TZP frameworks [16].

From an economic standpoint, processing pre-sintered Y-TZP discs offers additional advantages as it reduces milling time and tool (i.e., CAD/CAM) wear, thereby minimizing costs. However, after milling, pre-sintered Y-TZP frameworks must be sintered to achieve the desired mechanical properties, as stated by the manufacturer. Once this process is finalized, surfaces of zirconia frameworks are usually sandblasted and veneered with feldspathic porcelain [22]. It is worth noting that sandblasting can transform the tetragonal ZrO_2_ phase into a monoclinic phase, which can affect the mechanical properties and stability of Y-TZP materials [22].

Furthermore, the transformation to the monoclinic phase [2,6,7,16,24] can affect the mechanical properties and stability of Y-TZP materials [22]. The mechanical properties of Y-TZP rely on the aforesaid tetragonal to monoclinic transformation [5,23]. Interestingly, the tetragonal phase on the Y-TZP surface can transform into the monoclinic phase, which is accompanied by an increase in volume (4–5%) that induces compressive stresses [1,25]. Such transformation can yield severe clinical implications, such as an increased risk of fracture.

The structural properties of this type of material can be studied through the use of Powder X-ray Diffraction (PXRD) [26,27,28]. Furthermore, PXRD can be used to monitor the gradual change in t-ZrO_2_ and c-ZrO_2_ content that is governed by the Y^3+^ additions. It follows that this stabilizing Y^3+^ cation finely tunes Y-TZP properties such as translucence, toughness, and hardness. Thus, the Y^3+^ content in Y-TZP materials is especially important when using these ceramics for dental indications and restorations [1].

These dental ZrO_2_-based materials are characterized by both great toughness and strength. However, their great toughness has been associated with cracking issues [1,29,30,31]. The aforementioned studies also underscore that CAD/CAM processing can result in defects such as holes, cracks, and chipping. Therefore, it is of common interest to study the impact of processing (e.g., milling) on new dental materials to predict clinical implications such as an increased risk of fracture or accelerated aging. However, these issues can be alleviated to some extent by sintering [16,29].

Most often, only the flexural strength of individual layers is measured, whereas the overall mechanical performance of the multilayered Y-TZP systems is rarely investigated. This is crucial for the clinical application of strength-gradient multilayered Y-TZP material, as it must withstand the stresses encountered in various applications. To fully understand the properties of multilayered Y-TZP, the influence of different Y^3+^ contents on crystallographic structure, microstructure, and flexural strength should be investigated.

This study aimed to attain a better understanding of the mechanical properties and structure of diverse ZrO_2_ ceramic materials. The following properties, such as flexural strength, surface morphology, and phase composition, were characterized as they are important aspects for the success and durability of fixed prosthetic restorations in demanding clinical situations. This study involved three different ZrO_2_ (Y-TZP) materials that are commonly applied in dentistry. Overall, this study wants to test the null hypothesis, which means that we assume that there is no significant difference in the mechanical, morphological, and structural properties of diverse dental Y-TZP materials available on the market.

## 2. Materials and Methods

In this study, three types of different dental multilayered ZrO_2_-based (Y-TZP) materials were used (Table 1). For each material, i.e., I, S, and K, a group of *n* = 20 identical samples was prepared. The sample size was estimated using G-power analysis based on a preliminary study in which the same methodology and measuring instruments were used as in the research proposed. The mean flexural strength values were in the range of 650–880 MPa, and for the expected difference of at least 15% between the experimental groups, Cohen’s *d* = 1.17 was obtained, with a significance level of 0.05 and the assumption of an equal number of samples in all experimental groups results in a statistical power of 80% for *n* = 15 per experimental group. If the number of samples increases to *n* = 20 per experimental group, it is possible to obtain a statistical power greater than 95%. It should be noted that these I, S, and K materials have a similar composition and clinical application.

**Scheme 1:**
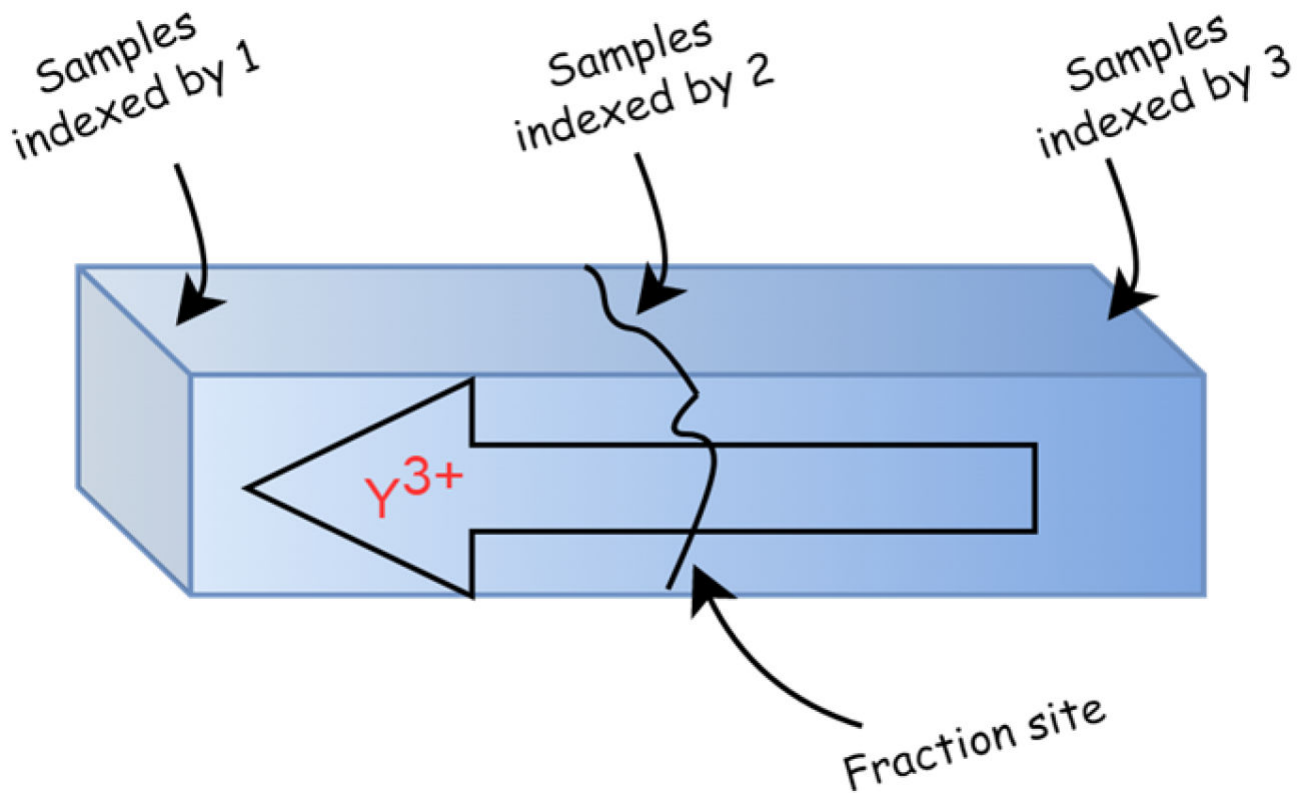
Schematic representation of Y-TZP bar samples prepared using CAD/CAM technology in this work. The content of Y^3+^ is increasing from the right (i.e., 3Y-TZP) to the left (i.e., 5Y-TZP). These bar samples are considered multilayers due to varying Y^3+^ amounts, which lead to changes in transparency and the materials’ mechanical, structural, and morphological properties.

Rectangular samples (i.e., bars) with dimensions of 2 mm × 2 mm × 16 mm (Scheme 1) were prepared from CAD/CAM discs of each material using the Programill PM7 (Ivoclar Vivadent AG) milling machine; the bars were constructed with a reduced thickness in comparison to standardized ISO specimens. Consequently, the bars were sintered according to the manufacturers’ instructions in Programat S1 1600 (Ivoclar Vivadent-Technical). IPS e.max ZirCAD Prime was sintered at a heating rate of 10 °C/min up to 900 °C and subsequent heating at 3.3 °C/min up to 1500 °C, followed by a dwell temperature and sintering time of 1500 °C for 2 h, and a cooling rate of 10 °C/min from 1500 °C to 900 °C and 8.3 °C/min from 900 °C to room temperature (in total 9 h 50 min). KATANA Zirconia YML was sintered at a heating rate of 10 °C/min from room temperature to 1550 °C, followed by a dwell temperature and sintering time of 1550 °C for 2 h, and a cooling rate of 10 °C/min from 1550 °C to room temperature (in total 7 h). Cercon ht ML was sintered at a heating rate of 11 °C/min from room temperature to 1500 °C, followed by a dwell temperature and sintering time of 1550 °C for 2 h, and a cooling rate of 11 °C/min from 1500 °C to room temperature. The samples were analyzed as received after the sintering, i.e., there was no additional processing after the sintering. According to Scheme 1, the bars are made of various multilayers containing different amounts of Y^3+^. The previous sentence aligns with the manufacturers’ data that does not specify the exact composition of S, I, and K materials.

**Scheme 2:**
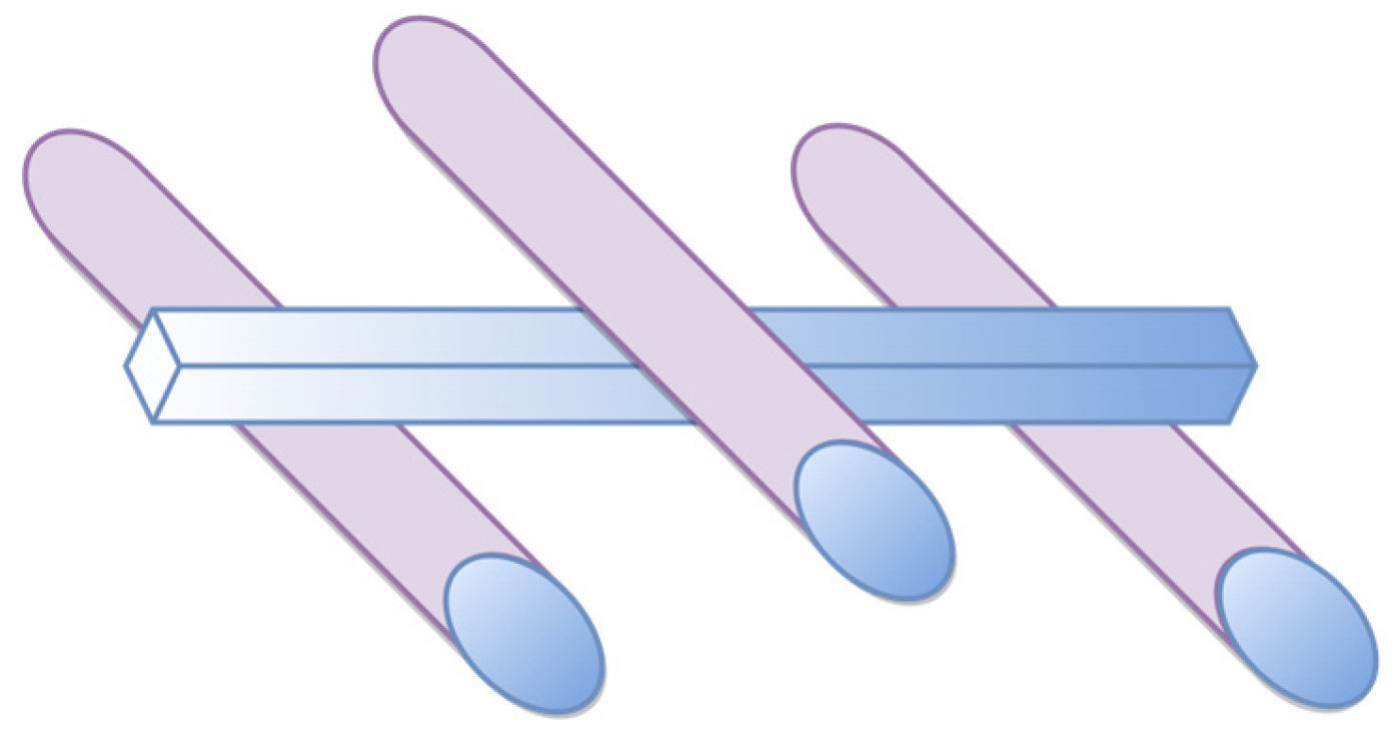
Schematic representation (inspired by [14]) of the three-point flexural strength test.

The prepared bars were loaded and tested until fracturing in the universal testing machine (Inspekt Duo 5kN; Hegewald & Peschke, Nossen, Germany) in a three-point flexural strength test (ISO 6872:2015) [32] with a 0.5 mm/min crosshead speed (Scheme 2). The bars were prepared to ensure that the top and bottom surfaces were put parallel to produce uniform contact with the two 5 mm-diameter rollers.

Rectangular cracked samples were mounted on metallic stubs and coated with a thin conductive layer of gold using Sputter Coater SC7620 for SEM evaluation at 10.000× magnification to assess the microstructures. Morphological (i.e., SEM) analysis was performed with an Axia ChemiSEM electron microscope (Thermo Fisher Scientific Inc., Waltham, MA, USA). 

The quantitative phase analysis of each material was performed using Powder X-ray Diffraction (PXRD), and the data were collected using the Bruker Discover D8 diffractometer supplied with a LYNXEYE XE-T detector (Karlsruhe, Germany). The measurements were taken in Bragg–Brentano geometry (1D) while applying CuK*α* radiation (1.54 Å) in the angular range 2θ from 10 to 70°. The step size was 0.02°, and a measuring time of 27 s/step was used. Rietveld structure refinement was performed using the HighScore Xpert Plus program 3.0 (Malvern Panalytical, Almelo, The Netherlands). Data were collected on the fracture edges of the samples that had previously been subjected to the three-point bending test.

The normality of distributions was checked by inspecting normal Q-Q plots and additionally verified with the Shapiro–Wilk test. Parametric statistics were used to compare flexural strength and flexural modulus among the three materials tested. The omnibus one-way ANOVA was followed by multiple comparisons among the materials using Tukey’s adjustment. Non-parametric equivalents of one-way ANOVA were employed in cases of significant deviations from normality. In addition to the comparison of mean values of the aforementioned three variables, Weibull statistics was used to evaluate the reliability of the materials by plotting the function: ln ln [1/(1 − Pf)] = m (ln σ − ln σθ), where Pf is the probability of failure, m is Weibull modulus, σ is flexural strength at failure, and σθ is characteristic strength. From this function, the parameter m; (Weibull modulus) was used as a measure of material reliability. All statistical analyses were performed at an overall significance level of 0.05. Statistical analysis was conducted using SPSS (version 25; IBM, Armonk, NY, USA), except for the Weibull analysis, which was performed using OriginPro (version 9.1; OriginLab, Northampton, MS, USA).

## 3. Results

### 3.1. Mechanical Properties

**Figure 1:**
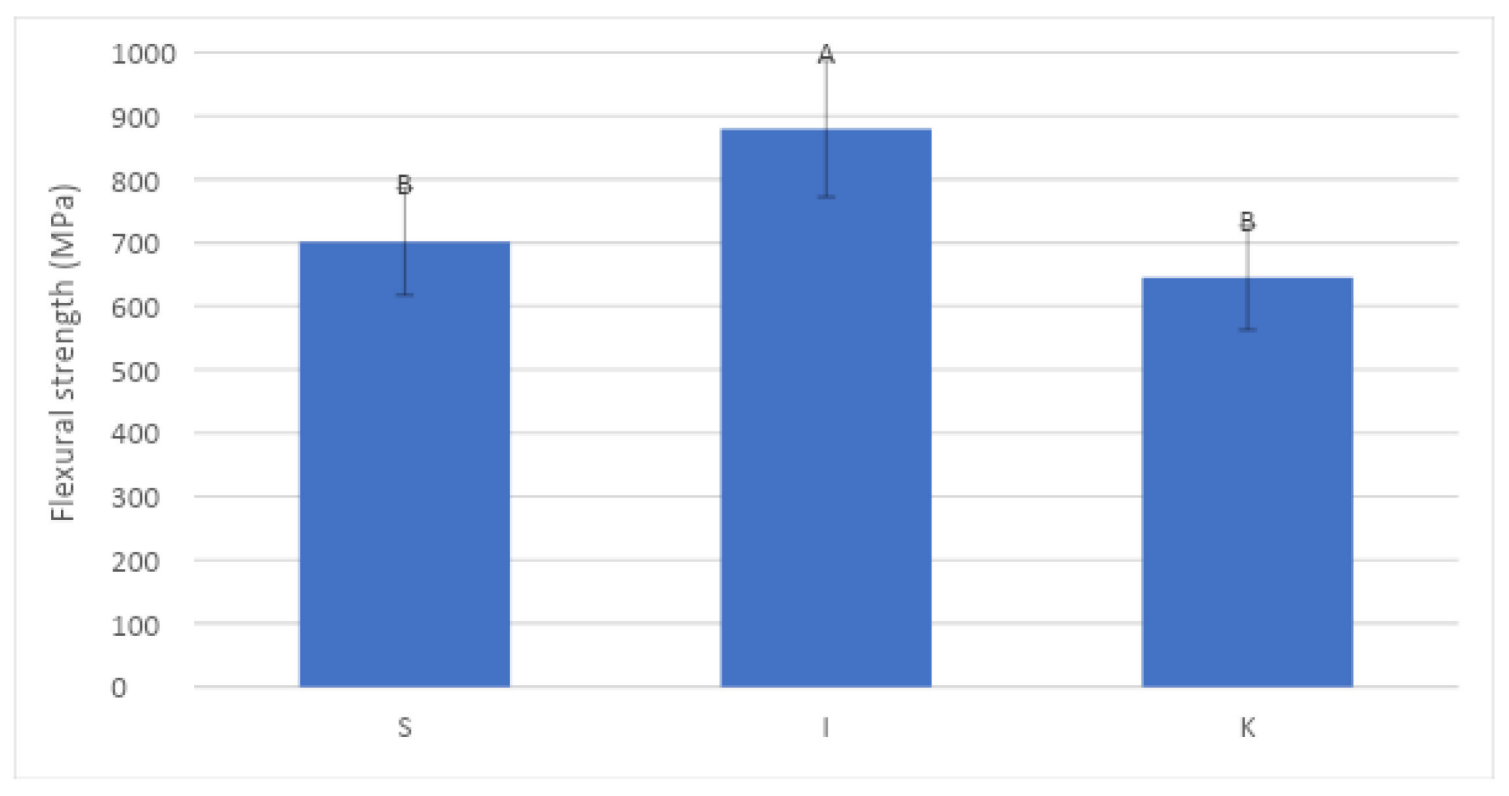
Mean flexural strength of the tested materials (A indicates a significant statistical difference from B).

One-way ANOVA showed that the material had a significant effect on flexural strength (*p* < 0.001). Tukey HSD post hoc test between paired groups revealed that the I material had notably higher flexural strength values compared to S and K materials (Figure 1). However, no particular difference was found in flexural strength between S and K materials.

The flexural strength values of the measured S and K bar samples were significantly lower (701.63 ± 84.21 and 644.73 ± 82.07 MPa) in comparison to sample I, which demonstrated the highest (879.42 ± 107.36 MPa) flexural strength value. These measurements are important as they describe the ability of a material to resist bending forces. Furthermore, the highest standard deviation (±107.36) was observed for the I sample, which can be attributed to the fact that this material possesses the highest flexural strength. The aforementioned observation points out that the I material should be more endurable in aggressive conditions that are present in the oral cavity.

**Figure 2:**
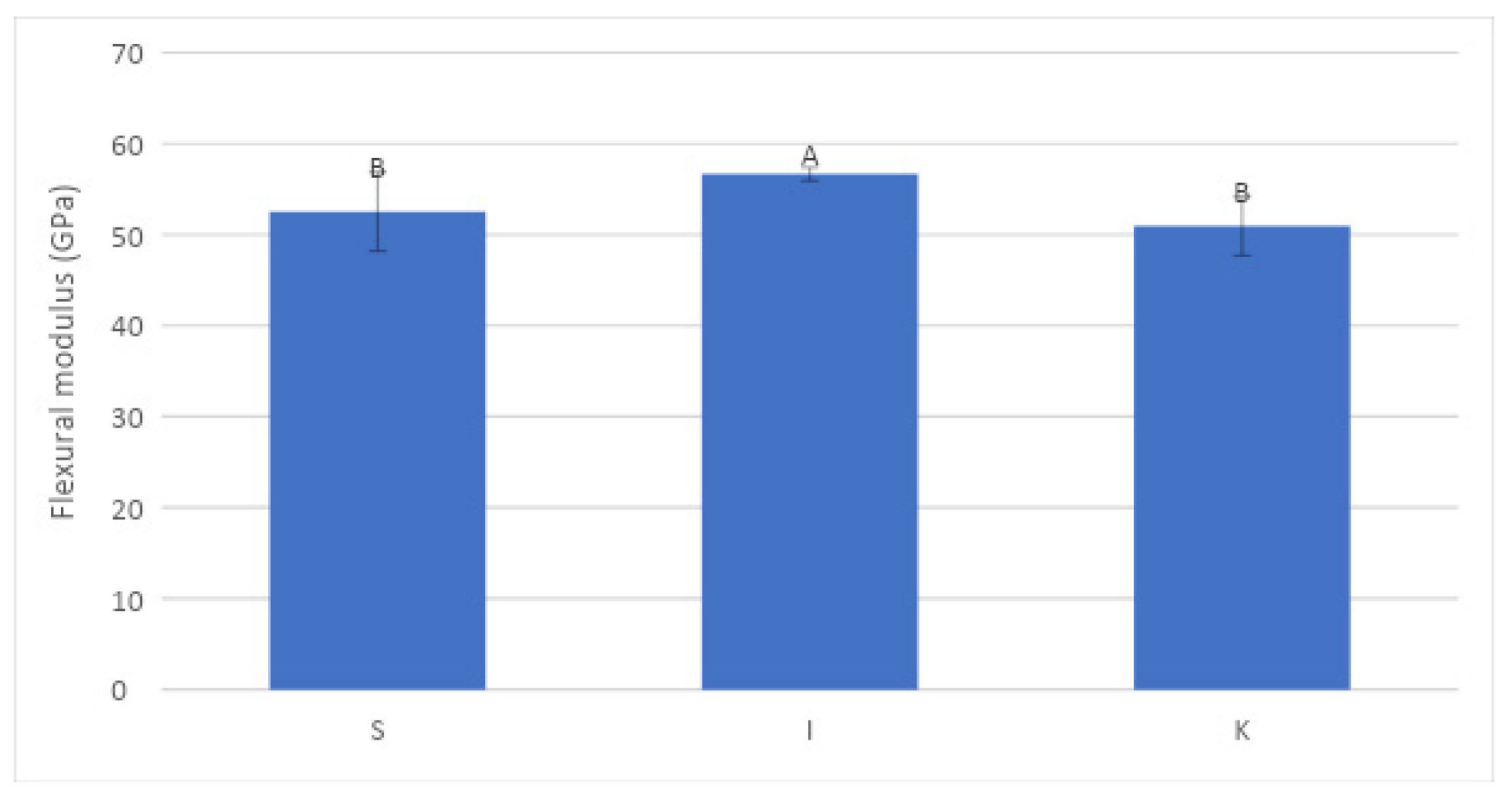
Mean flexural modulus (GPa) of the tested materials (A indicates a significant statistical difference from B).

Next, the flexural modulus, which defines the tendency of a material to bend under the applied force, was also investigated. A high flexural modulus value indicates that the material is stiff and resistant to bending. Herein, one-way ANOVA showed significant differences among the investigated I, S, and K materials, as demonstrated in Figure 2. Tukey’s HSD post hoc test between the paired groups (each group consisted of 20 samples) showed that S and K samples had a significantly lower flexural modulus, indicating the higher flexibility of sample I in contrast to each of the other samples (*p* < 0.001) (Figure 2).

According to Figure 2, the highest flexural modulus was measured for sample I (56.64 ± 0.73 GPa), while samples S and K (52.52 ± 4.34 and 50.93 ± 3.22 GPa) showed somewhat lower values. This observation leads us to the conclusion that sample I is stiffer. However, keep in mind that an increased dental material stiffness and brittleness can facilitate crack propagation, which may result in subsequent fractures and/or accelerated aging. On the other hand, an increased stiffness would improve the load spreading, which is an important aspect when dealing with dental materials.

**Figure 3:**
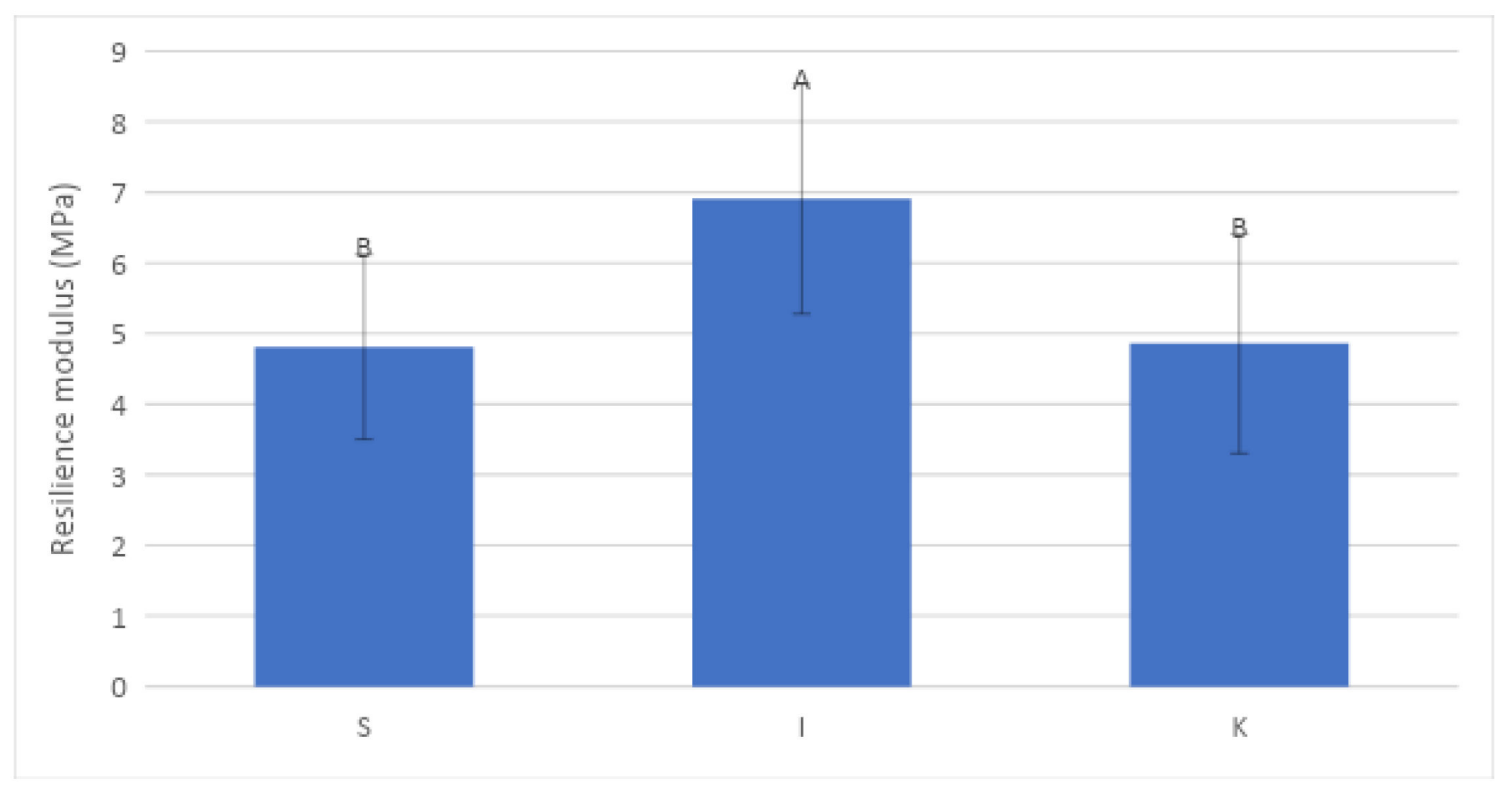
Mean resilience modulus (RM) of the tested materials (A indicates significant statistical difference from B).

Furthermore, from the data presented in Figure 1 and Figure 2, it is possible to compute the resilience modulus of each S, I, and K material. The calculated resilience modulus values are presented in Figure 3, and it can be observed that one sample (I) stands out in particular. Generally, the resilience modulus values can also be correlated to the density and porosity of the material [33]; and thus, this modulus is especially important when forecasting the material’s functional durability in the oral cavity.

The modulus of resilience (RM) for sample I (6.91 ± 1.63) showed the highest value compared to other materials, while materials S (4.81 ± 1.31) and K (4.86 ± 1.56) yielded similar RM values. The greater RM values indicate a higher ability to absorb more energy per unit volume without creating a permanent distortion. Additionally, an increased RM value often indicates lower porosity, which, in turn, should hinder both diffusion and accelerated aging. Taking into account the aforesaid arguments regarding resilience modulus, it is fair to say that the material I is more suitable for dental applications. 

### 3.2. SEM Microstructural Analysis

The qualitative SEM analysis of the tested materials showed the microstructural features of each S, I, and K material. These bar samples were examined using SEM after cracking, and we analyzed the positions with the highest and lowest portion of Y^3+^ as well as the fracture site. To be more precise, the bar site with the highest Y^3+^ content was indexed by 1 (S1, I1, and K1), while the site with the lowest Y^3+^ portion was labeled by 3 (S3, I3, and K3) (see Table 2 and Scheme 1). The positions at which the samples fractured during the examination are designated as S2, I2, and K2 (Table 2). 

**Figure 4:**
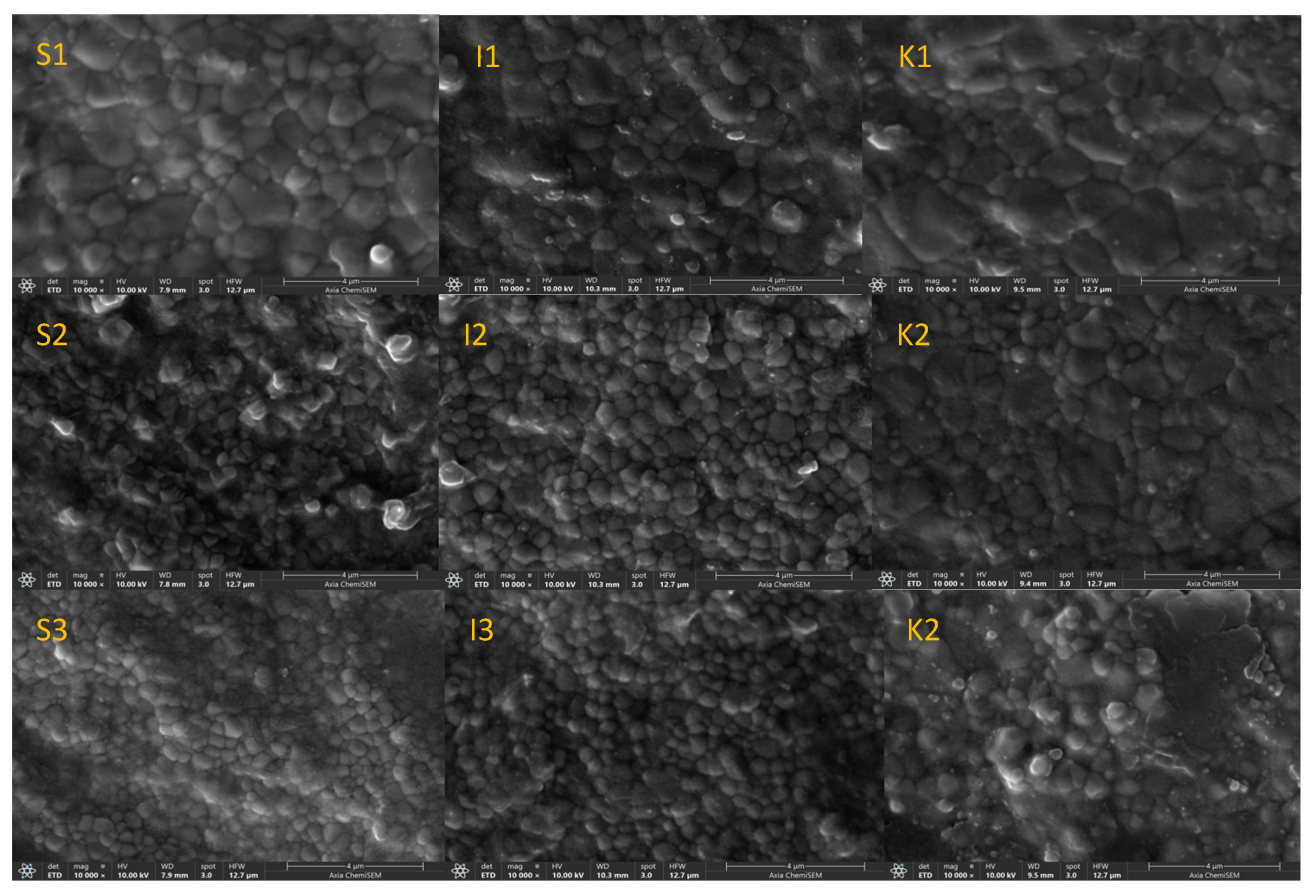
SEM images (×10,000) of monolithic CAD/CAM materials S (S1–S3); I (I1–I3), and K (K1–K3). The images show microstructures and grains of 5Y-TZP (indexed by “1”), 3Y-TZP (indexed by “3”), and microstructures of the fracture sites (indexed by “2”).

Figure 4 shows the SEM images (×10.000) of the 3Y-TZP (S3, I3, and K3) and 5Y-TZP (S1, I1, and K1) samples. One can observe that samples S1, I1, and K1 are composed of grains that are greater in size than the ones in the S3, I3, and K3 samples. Furthermore, the morphology of samples I2 and S2 (i.e., the point of fracture) is rather similar to that of I3 and S3, and there are no drastic changes in the grain size. On the other hand, samples K2 and K3 show grains of different dimensions, which indicates a difference in composition, i.e., a difference in Y^3+^ content. Additionally, it can be noted that the K2 sample shows a greater grain size than sample I2, which suggests that these samples were fractured at positions with different ratios of cubic and tetragonal phases (Figure 4).

**Scheme 3:**
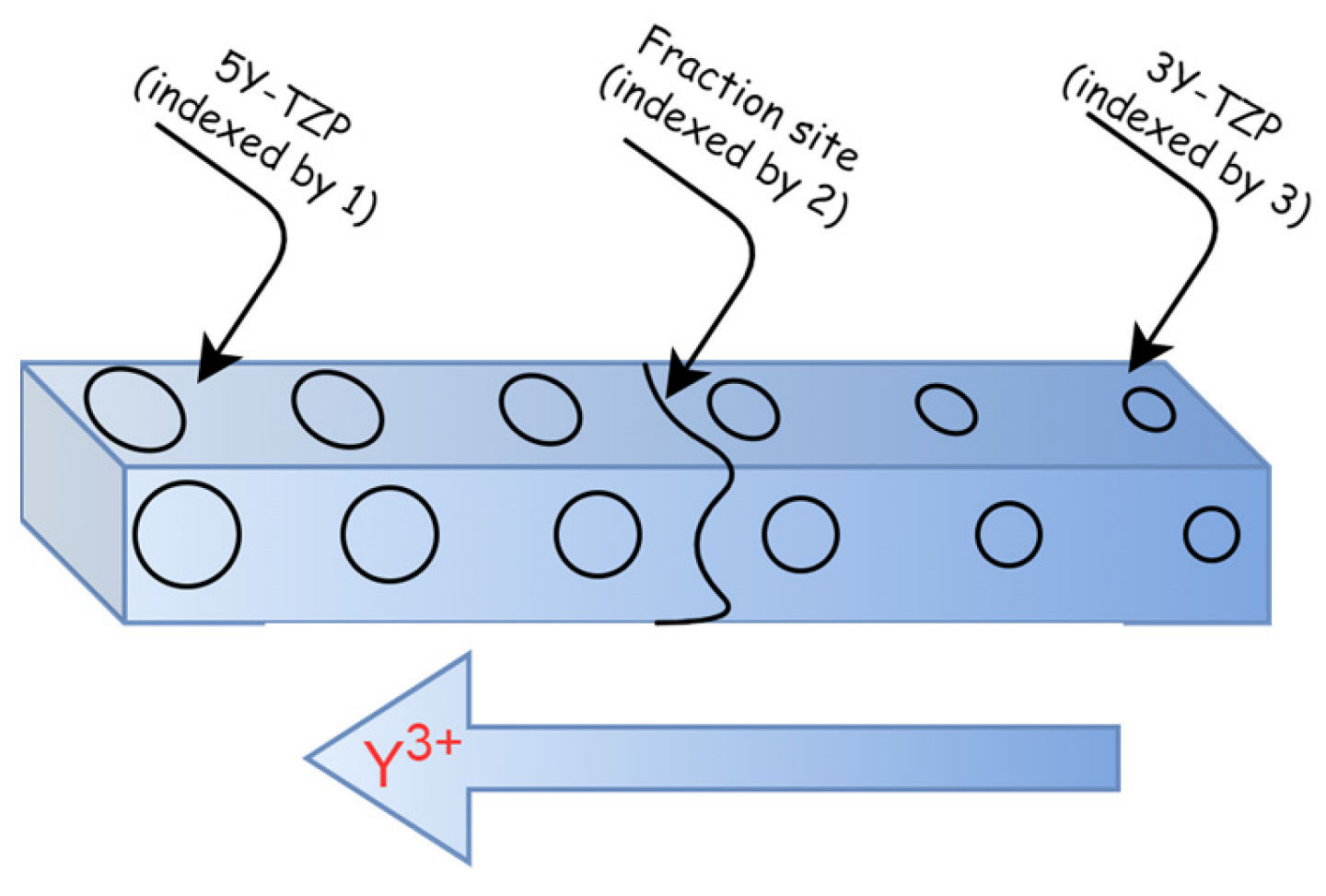
Schematic representation of bar samples used in this work. The grain sizes are arbitrarily chosen for illustration purposes. The scheme depicts a trend of increasing grain size alongside Y^3+^ content, which correlates with the trend presented in Figure 4. According to manufacturers, the size of the grains is governed by the Y^3+^ content; however, the exact layer composition is not designated.

Generally, as illustrated in Figure 4, the grain size of each sample steadily increases with increasing Y^3+^ content. This effect is shown in detail in Scheme 3. Notably, the samples indexed by 3 have the lowest Y^3+^ content and the smallest grain size. On the other hand, the samples indexed by 1 have the highest Y^3+^ amount and grain sizes. Most importantly, Scheme 3 suggests that each of the bar layers (milled from S, I, and K materials) has a different morphology and chemical composition that was determined by the manufacturer.

### 3.3. PXRD Quantitative Phase Analysis

PXRD data were collected on the edges (indexes 1 and 3) and on the fracture sites (index 2) of the S, I, and K samples that were previously subjected to the three-point bending test (Table 3). PXRD was employed for both phase identification and calculation of the relative cubic zirconia (c-ZrO_2_) and tetragonal zirconia (t-ZrO_2_) phase content (Table 3). Phase identification confirmed that all manufacturers use 5Y-TZP (higher Y^3+^ content) for enamel layers, while the dentine layers consist of 3Y-TZP (lower Y^3+^ addition). Additionally, PXRD showed that the intermediate layers include individual mixtures of 3Y-TZP and 5Y-TZP. Overall, the PXRD results show that the higher Y^3+^ content increases the amount of the cubic phase and consequently reduces the amount of the tetragonal phase. Note that only the compositions of the fracture sites of samples S2 and I2 are similar to the compositions of S3 and I3 (i.e., 3Y-TZP).

Quantitative PXRD analysis revealed that K1 and S1 contained a similar amount (60.8 and 56.4%) and the highest amount of c-ZrO_2_ phase (5Y-TZP), whereas I3 contained the highest amount of t-ZrO_2_ phase (3Y-TZP). The phase composition of K2 (i.e., the point of fracture) had a comparable t-ZrO_2_ and c-ZrO_2_ phase composition (47.3 and 52.7%). In samples I2 and I3, the t-ZrO_2_ phase content was almost identical. Additionally, similar t-ZrO_2_ content can be found in samples S2 and S3. These observations point out that the fracture I2 (and S2) site had the same composition as the dentin layer of the I3 (and in S3) samples.

## 4. Discussion

The objective of this comparative in vitro study was to evaluate the mechanical properties of three different multilayer zirconia ceramics: ZirCAD Prime (I), CERCON ht ML (S), and Katana ZIRCONIA YML (K). The flexural strength and resilience modulus were measured for each material, and the microstructure and phase composition were analyzed using SEM and Rietveld refinement, respectively. As the material properties studied herein exhibited different mechanical, morphological, and structural properties, our null hypothesis can be rejected.

The highest flexural strength was recorded for I, followed by S and K materials. The presented results indicate that as the t-ZrO_2_ content increases, its flexural strength increases. Thus, our findings are per the previous studies that have also shown that higher t-ZrO_2_ phase content leads to higher flexural strength in Y-TZP ceramics. [34,35]. 

An increase in the stabilizing Y_2_O_3_ oxide yields a higher c-ZnO_2_ content, which can reduce the flexural strength of the material [36]. Thus, we have compared the three multilayer zirconia ceramics in this study, and the results revealed statistically significant differences in flexural strength values (Figure 1). Furthermore, Table 3 shows that the Y-TZP phase composition is altered by Y^3+^ addition. Nevertheless, the flexural strength of the I material is greater than that of the S and K materials, suggesting that both the processing and composition of each material are important factors [37,38,39].

ZirCAD Prime demonstrated the highest overall flexural strength of 879.42 MPa due to the highest t-ZrO_2_ content. However, the 879.42 MPa value is less than the 1200 MPa value stated by the manufacturer (i.e., Ivoclar Vivadent, 2019). The 1200 MPa value was likely determined for the body dentin layer (i.e., 3Z-TZP) using a biaxial flexural strength test, which typically yields higher flexural strength values than the three-point test that was employed in this study [40]. In three-point bending tests, bar-shaped samples were loaded centrally in such a way that the maximal tensile stress was concentrated on the bottom of the bars and between the edges. These areas frequently contain flaws, which may occur during the CAD/CAM manufacturing of the samples. Such flaws can promote crack formation and, ultimately, specimen failure.

Generally, data typical for this study depend on a variety of variables, including the experimental set-up, testing parameters, and the test procedure [41]. Milling and surface preparation have a particularly strong impact on the flexural strength of the tested ceramic materials, which are already weakened by the surface damage caused by the milling process [42]. Furthermore, Inokoshi et al. [43] found that the harmony between microcrack layout and surface compressive pressure, resulting from tetragonal to monoclinic phase transformation, controls the strength of zirconia after Al_2_O_3_-sandblasting [43]. If these facts are taken into account, it is clear that data variation (e.g., standard deviations) in this study was caused by the impact of different variables.

Furthermore, the elastic modulus and resilience values for samples S and K were significantly lower than those for the I sample, indicating that these samples are less flexible and stiff. The higher resilience modulus indicates a higher ability to absorb more energy per unit volume without creating a permanent distortion, whereas the flexural modulus defines the tendency of a material to bend under an applied force. The increased flexibility and resilience of the I material can be observed in the results of the failure mode evaluation. The combination of the increased flexibility and stiffness observed for the I sample in this work makes this material an appropriate choice for crown fabrication.

In the present study, microstructure analysis and zirconia phase quantification revealed differences in grain size and phase content across material layers. In a direct comparison of images (Figure 4) from various materials, the grain size and cubic phase content gradually decrease from the enamel (5Y-TZP) to the dentin (3Y-TZP) layer (Table 3). The 5Y-TZP and its adjacent transition zone have a higher cubic phase content and a larger average grain size than the 3Y-TZP. 5Y-TZP generally showed the largest grains, which agrees with findings from other studies [44,45]. For all the investigated S, I, and K materials, the grain size of 3Y-TZP (vs. 5Y-TZP) was lower.

The intermediate layers between 3Y-TZP (S3, I3, and K3) and the point of the crack (S2, I2, and K2) showed a similar morphology consisting of smaller and larger grains (Figure 4). The larger grains that are typical for 5Y-TZP (S1, I1, and K1) were also visible at the point of the crack, indicating that the interconnected zones of the 3Y-TZP and 5Y-TZP materials traverse the layers (indexed by 1, 2, and 3). These findings are consistent with the information provided by the manufacturer, which states that the enamel layer is composed of 5Y-TZP, and the dentin layer is 3Y-TZP [46]. 

The strength variation of the investigated Y-TZP materials is attributed to the varying Y^3+^ concentrations across the layers, leading to distinct Y-TZP phase compositions (Table 3). Rietveld refinement revealed an increase in the c-ZrO_2_ phase from the dentine to enamel layers (Table 3), which agrees with previous studies [46,47]. The 3Y-TZP samples (i.e., S3, I3, and K3) exhibited the highest portion of the t-ZrO_2_ phase, while the 5Y-TZP layers (S1, I1, and K1) embodied the least. 

Intriguingly, material I showed the highest amount of tetragonal phase (Table 3), the smallest grain size (Figure 4), and the highest flexural strength (Figure 2). Consistent with prior findings, these results support the correlation between Y^3+^ content and flexural strength. These findings align with previous studies [48,49,50] reporting that the variation in Y^3+^ content led to different c-ZrO_2_ and t-ZrO_2_ phase compositions and, concomitantly, a difference in flexural strength. 

The quantitative phase composition analysis revealed that all Y-TZP samples (Table 3) mostly contained the t-ZrO_2_ phase (samples indexed by 2 and 3). All of the samples were sintered to achieve the stable t- and c-ZrO_2_ phase composition in Y-TZP samples obtained through Y^3+^ addition. The stable multilayer composition of Y-TZP materials (Table 1) was responsible for the increase in global residual compressive stresses during the sintering process, which resulted in increased crack inhibition, fracture resistance, and zirconia flexural strength. The closing described above is consistent with other studies [51,52,53].

## 5. Conclusions

The findings in this work demonstrated that the multilayer dental Y-TZP materials exhibited different mechanical and morphological properties. This variation in properties is expected due to the differences in their compositions since they are sourced from various manufacturers.

It was observed that the diverse dental materials used in this work consisted of different amounts of t-ZrO_2_ and c-ZrO_2_ phases, which resulted in grains of various sizes. However, the I sample with the highest flexural strength contained grains of uniform size at the point of fraction. Consequently, it can be concluded that the presence of a homogeneous grain size distribution exerted a significant positive effect on the flexural strength of multilayer Y-TZP material. 

The distribution of Y^3+^ in the tested multilayer zirconia samples was quite different, i.e., the dentin layer had the lowest Y^3+^ concentration while the enamel layer had the highest Y^3+^ content, whereas the Y^3+^ amount in the transition layer was in between. It was presented that the fraction site of the **I** sample had the highest portion of the t-ZrO_2_ phase, which resulted in the highest flexural strength value.

This research also revealed that morphological data from SEM images, as well as the resilience modulus and flexural strength values, can all be used to forecast the material’s functional durability. Finally, it was elaborated that these data can be used to assess the material’s porosity and cracking potential, both of which directly affect the aging process.

## Figures and Tables

**Table 1:** List of different samples applied in this study.

Material Name	Manufacturer	Sample Name	Flexural Strength	LOT Number
ZirCAD Prime	Ivoclar Vivadent (Schaan, Liechtenstein)	I	1200 MPa	Z05RMR
Cercon ht ML	Dentsply Sirona (Charlotte, NC, USA)	S	1200 MPa	0018041712-1057
Katana ZIRCONIA YML	Kuraray Noritake (Tokyo, Japan)	K	1100 MPa	EJHNA

**Table 2:** Name of the samples applied in this study. The amount of Y^3+^ for each sample is increasing from right to left. Samples indexed by 1 (S1, I1, and K1) represent the layer with the highest Y^3+^ content (5Y-TZP), whilst the samples indexed by 3 (S3, I3, and K3) are characterized by the lowest Y^3+^ addition (3Y-TZP). Samples labeled by 2 represent the fraction position.

Sample	5Y-TZP	Fracture Site	3Y-TZP
	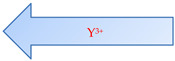		
S (Sirona)	S1	S2	S3
I (Ivoclar)	I1	I2	I3
K (Katana)	K1	K2	K3

**Table 3:** Quantitative phase analysis of Samples S1, S2, S3, I1, I2, I3, K1, K2, and K3.

**Sample**	**Phase**	**Sample Index**		
		**5Y-TZP**	**Fracture Site**	**3Y-TZP**
		1	2	3
		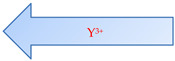		
S	c-ZrO_2_ (wt %)	56.4	22.8	22.5
	t-ZrO_2_ (wt %)	43.6	77.2	77.5
I	c-ZrO_2_ (wt %)	50.1	13.5	13.9
	t-ZrO_2_ (wt %)	49.9	86.5	86.1
K	c-ZrO_2_ (wt %)	60.8	47.3	31.6
	t-ZrO_2_ (wt %)	39.2	52.7	68.4

## Data Availability

Data are contained within the article.
